# Risk of Valproic Acid-Related Tremor: A Systematic Review and Meta-Analysis

**DOI:** 10.3389/fneur.2020.576579

**Published:** 2020-12-15

**Authors:** Chen qi Zhang, Bao ming He, Mei ling Hu, Hong bin Sun

**Affiliations:** Department of Neurology, Sichuan Academy of Medical Sciences and Sichuan Provincial People's Hospital, Chengdu, China

**Keywords:** valproic aid, tremor, random control trials, meta-analysis, systematic reveiw

## Abstract

**Purpose:** To evaluate the incidence and risk of tremor in patients treated with valproic aid (VPA) monotherapy.

**Methods:** We searched the PubMed, Embase, and Cochrane Library databases to gather relevant data on tremor in patients taking VPA and other drugs and performed a meta-analysis using Stata15.1 software.

**Results:** Twenty-nine randomized controlled trials (RCTs) met the inclusion criteria and were included in the meta-analysis. The overall incidence of tremor in patients receiving VPA therapy was 14% [OR = 0.14, 95% CI (0.10–0.17)]. The pooled estimate risk of tremor showed a significant difference between patients treated with VPA and all other drugs [OR = 5.40, 95% CI (3.22–9.08)], other antiepileptic drugs (AEDs) [OR = 5.78, 95% CI (3.18–10.50)], and other non-AEDs [OR = 4.77, 95% CI (1.55–14.72)]. Both a dose of <1,500 mg/d of VPA [included 500 mg/d: OR = 3.57, 95% CI (1.24–10.26), 500–999 mg/d: OR = 3.99, 95% CI (1.95–8.20), 1,000–1,499 mg/d: OR = 8.82, 95% CI (3.25–23.94)] and a VPA treatment duration of <12 m [included ≤ 3 months: OR = 3.06, 95% CI (1.16–8.09), 3–6 months: OR = 16.98, 95% CI (9.14–31.57), and 6–12 months: OR = 4.15, 95% CI (2.74–6.29)] led to a higher risk of tremor than did other drugs, as did higher doses and longer treatment times.

**Conclusion:** Compared with other drugs, VPA led to a higher risk of tremor, and the level of risk was associated with the dose and duration of treatment.

## Introduction

VPA (valproic acid), a clear, colorless, eight-carbon branched-chain fatty acid, was first produced in 1882 as an organic solvent (1). Then, in 1963, the therapeutic potential of VPA was fortuitously discovered by Carraz et al. (2), who recognized that VPA itself has anticonvulsant properties. VPA has been approved by the Food and Drug Administration (FDA) and is the first-generation broad-spectrum antiepileptic drug (AED) most commonly administered to treat generalized and focal epilepsies in children and adults, and it is also used to treat bipolar disorder, posttraumatic stress disorder (PTSD), schizophrenia, neuropathic pain, and migraine headaches (3–7). It may be useful in novel applications that are currently being researched, such as cancer therapy and prevention (8). Despite its utility, VPA is associated with several common side effects, including tremors, weight gain, alopecia, liver dysfunction, gastrointestinal disturbances, increased triglyceride levels, thrombocytopenia, etc. (9–12).

Tremor, one of the most common neurological symptoms, is defined as an involuntary, rhythmical, oscillatory movement of a body part produced by either synchronous or alternating contractions of antagonist muscles (13). Tremor is the most common side effect involving the central nervous system, occurring in as many as one quarter of chronically treated patients (14, 15). VPA-related tremors are usually action or postural tremors, but sometimes they are rest tremors (16). Tremors do not usually abate with continued treatment (10), so we investigated and diagnosed with the utmost caution, but in some cases, tremors may respond to smaller dosages or changes in the dosing regimen. Additionally, tremors also occur relatively commonly in patients taking antipsychotics, antidepressants, sympathomimetics, antiarrhythmics, AEDs, and other drugs (14, 17), such as lithium, phenytoin (PHT), carbamazepine (CBZ), topiramate (TPM), vigabatrin (VGT), lamotrigine (LTG), and gabapentin (TGB).

However, the relationship between VPA dosage and tremor is currently unclear. In a study by Karas et al., tremors usually appeared with dosages >750 mg per day (16). Patients exhibit tremors with doses exceeding 1,000 mg/d, according to Hyman's reports (18). Additionally, trials have shown that patients with doses of <500 mg also experience tremor (19, 20). In addition, it is not clear whether the dose of VPA compared to other drugs increases the risk of tremors. At present, there is relatively little information about VPA-associated tremors in the existing medical literature, and most of the existing studies are limited to studies with small sample sizes or case reports. To provide more evidence about the incidence of VPA-associated tremor as well as compare the risk of tremor between VPA and other drugs, we systematically reviewed related articles and analyzed them.

## Methods

Our study did not require ethical approval because patients were not involved. According to the PRISMA (21) principles and MOOSE (22) guidelines, the search strategy, selection criteria, data extraction process, quality assessment, and statistical analysis were predesigned based on the Cochrane Review Methods. The protocol was not registered on any website.

### Search Strategy

Two researchers searched online databases, including the PubMed (1977 to February 2, 2020), Embase (1982 to February 2, 2020), and Cochrane Library databases (2001 to February 2, 2020), for potentially relevant studies; there were no language restrictions, but the search was restricted to human studies. The search was conducted with a combination of medical subject headings and term words, including “Valproic acid”[Mesh], “Propylisopropylacetic Acid,” “2-propylpentanoic acid,” “Divalproex,” “Depakene,” “Divalproex Sodium,” “Valproate,” “Valproate Sodium,” “Valproate Calcium,” “VPA,” “Depakine,” “Depakote,” “tremor” [Mesh], “Tremor,” “Tremors,” and “drug-induced tremor.” Additionally, the references of all the included studies or related reviews were screened to avoid accidental omissions.

### Selection Criteria

Clinical trials that met the following criteria were included: (1) the design of the trial was an RCT (randomized controlled trial), (2) VPA was the only therapy provided to the control group or test group, and (3) the study provided the original data for VPA-associated tremor and comparisons with the control group. The exclusion criteria were as follows: (1) observational studies (including cross-sectional studies, case–control studies, cohort analyses, and so on), (2) studies in which VPA was combined with one or more other drugs and compared with a control group, (3) comparative studies with two different valproate preparations, (4) studies in which VPA-associated tremor was not mentioned or the data provided were incomplete, and (5) studies based on the same study population. According to the inclusion and exclusion criteria, we identified a total of 29 randomized controlled trials (six studies were placebo-controlled studies).

### Data Extraction

Two reviewers independently extracted relevant information from each eligible study, and any discrepancies between the two reviewers were resolved by consulting with the senior author. We established a data extraction form (Table 1), which included the first author's name, publication year, study recruitment method, study field, design, whether the trial include one or multiple centers, sample sizes of the test group and control group, median age of the participants, percentage of females, dose of VPA, duration of treatment, and number of adverse outcomes related to tremor.

**Table 1:** Characteristics of included studies.

**Study name**	**Year**	**Recrui-tment**	**Study field**	**Design**	**Multi/Single center**	**No. randomized**	**No. analysis**	**Drug**	**Age (range/mean)**	**Gender (female %)**	**VPA does (mean mg/d)**	**No. tremor (control/VPA)**	**Incidence of tremor**	**Duration time**
Blumenfeld	2008	USA	MA	RCT	Single	59	30/29	BoNTA/VPA	18–65 y (42.4 ± 10.3)	84.70%	500	0/3	0.10	10.5 m
Calabrese	2005	USA	BD	RCT	Single	60	32/28	Lithium/VPA	≥18 year (37 ± 8.2)	51.70%	1,571	9/1	0.04	20 m
Christe	1997	multi-nation	EP	RCT	Multi	249	128/121	OXC/VPA	15–65 y	49%	1,146.2	2/19	0.16	45 m
Craig	1994	UK	EP	RCT/ Single blind	Not clear	42	25/17	PHT/VPA	62–88 y (77)	Not state	688	0/5	0.29	1 y
Hebrani	2009	Iran	BD	RCT	Single	120	59/61	TPM/VPA	12–18 y	60%	1,200	20/14	0.24	8 w
Hesami	2018	Iran	MA	RCT	Single	82	46/36	Atorvastatin/VPA	18–50 y (33.25 ± 9.91)	96.30%	500	1/4	0.11	3 m
Mathew	1995	USA	MA	RCT	Multi	105	36/69	placebo/VPA	16–75 y	80%	1,087	0/9	0.13	16 w
Nejad	2009	Iran	EP	RCT/ Open label	Single	46	23/23	LTG/VPA	8–30 y	Not clear	800	1/4	0.17	28 w
Park	2013	Korea	EP	RCT/ Open label	Single	33	16/17	TPM/VPA	13–36 y	52%	1,200	0/4	0.24	32 w
Privitera	2003	Multi-nation	EP	RCT	Multi	613	409/126/78	TPM/CBZ/VPA	≥6 year	71.80%	1,250 mg	4/2/17	0.22	6 m
Biton	2001	USA	EP	RCT	Multi	133	65/68	LTG/VPA	12–76 y (30.1 ± 14)	56.40%	1,822 ± 633	2/19	0.28	24 w
Mattson	1992	USA	EP	RCT	Multi	480	236/244	CBZ/VPA	18–70 y	7%	2,099 ± 824 mg	27/77	0.32	12 m
Afshari	2012	Iran	MA	RCT	Single	56	28/28	TPM/VPA	18–65 y	28(50%)	400 mg	4/6	0.21	12 w
Bostani	2013	Iran	MA	RCT	Single	104	50/54	Cinnarizine/VPA	Adults (31.85 ± 7.76)	68.30%	400 mg	0/8	0.14	12 w
Klapper	1997	USA	MA	RCT	Multi	137	44/45	Palcebo/VPA	17–65 y (40.8)	93%	500 mg	0/0	0.00	12 w
Klapper	1997	USA	MA	RCT	Multi	137	44/43	palcebo/VPA	21–70 y (41.5)	88%	1,000 mg	0/7	0.16	12 w
Klapper	1997	USA	MA	RCT	Multi	137	44/44	palcebo/VPA	23–76 y (40.7)	84%	1,500 mg	0/16	0.36	12 w
Fakhoury	2004	USA	EP	RCT/ Open label	Multi	158	105/53	LTG/VPA	≥16year	41%	Not state	5/11	0.21	28 w
Richens	1994	USA	EP	RCT/ Open label	Multi	300	126/174	CBZ/VPA	≥16 year	Not clear	924	3/9	0.05	3 y
Sarchielli	2014	Italy	MA	RCT	Multi	88	44/44	placebo/VPA	18–65y	77.30%	800	0/1	0.02	6 m
Silberstein	1999	MA	MA	RCT	Multi	163	46/117	placebo/VPA	19–68 y	85%	1,000	0/23	0.20	6 m
So	1992	USA	EP	RCT	Not clear	33	17/16	CBZ/VPA	10–70 y	56.30%	585 umol/l	0/4	0.25	24 w
Sobaniec	2004	Poland	EP	RCT	Multi	42	19/23	Carbatrol/VPA	18–52 y (30.8 ± 7.92)	21%	730	0/2	0.09	8 w
Steinhoff	2005	Germany	EP	RCT/ Open label	Multi	239	88/121/30	CBZ/LTG/VPA	≥12 year	53.30%	1,050	0/0/3	0.10	24 w
Stephen	2007	Germany	EP	RCT/ Open label	Single	225	114/111	LTG/VPA	13–80 y	44.10%	1,000 mg	0/3	0.03	3 m
Tabrizi	2019	Iran	EP	RCT/ Open label	Single	103	45/58	LEV/VPA	≥16 year (29 ± 9.7)	29.30%	1,000 mg	0/1	0.02	26 w
Togha	2008	Iran	MA	RCT	Not clear	125	67/58	Cinnarizine/VPA	16–60 y	77.60%	600 mg	Not clear/3	0.05	12 w
Tohen	2008	multi-nation	MA	RCT	Multi	521	105/215/201	Placebo/ Olanzapine/VPA	≥16 year (40.6 ± 12.8)	46.50%	848.4 ± 135.62	0/4/10	0.05	3 w
Wheless	2004	USA	EP	RCT	Multi	119	23/77/19	CBZ/TPM/VPA	6–16 year	58.00%	1,250	1/1/2	0.11	6 m
Xu	2015	China	BD	RCT	Single	114	39/37	Olanzapine/VPA	20–60 y (30.7 ± 7.8)	91.80%	1,530 ± 220	3/0	0.08	4 w
Yurekli	2008	Turkey	MA	RCT	Single	70	30/40	placebo/VPA	14–76 (40 ± 14.5)	87.50%	1,000 mg	0/1	0.03	3 w

*EP, epilepsy; BD, bipolar disorder; MA, migraine headache; RCT, randomized controlled trial; VPA, Valproic acid; LTG, Lamotrigine; CBZ, Carbamazepine; OXC, oxcarbazepine; TPM, topiramate; PHT, phenytoin; LEV, levetiracetam; m, month; y, year, w, week*.

### Quality Assessment

The quality of the RCTs was assessed by two authors, and any disagreements were resolved via discussion with a third reviewer, after which the trial was reevaluated. The guidelines for assessing risk of bias provided in the Cochrane handbooks http://community.cochrane.org/handbook were used to assess the quality of the included studies, and the domains assessed included random sequence generation, allocation concealment, blinding of the participants and personnel, blinding of the assessors, incomplete outcome data, and selective outcome reporting.

### Statistical Analysis

We performed statistical analyses using Stata 15.1 software. We calculated the odds ratios (ORs) and 95% confidence intervals (CIs) to demonstrate the pooled effects of tremor with VPA compared to other drugs. Forest plots were used to visually display the meta-analysis results. We used fixed-effect models to weight the studies by the amount of available information, whereas a random-effect model was used for heterogeneity between studies, and Cochran's Q statistic and I^2^ metric statistics were used to assess the level of heterogeneity. For the Cochran Q test, heterogeneity was considered statistically significant when *P* < 0.05. For I^2^, if I^2^ > 50%, the level of heterogeneity was considered unacceptable, and the data were analyzed with a random-effect model. A fixed-effect model was applied when I^2^ < 50%. *P* < 0.05 indicated statistical significance, and all tests were two-sided. In our study, we performed analyses of different control groups [AEDs (excluding VPA) and other non-AEDs]. Obvious heterogeneity was assessed by subgroups based on the sample size, VPA dose, and treatment duration.

## Results

### Included Studies

**Figure 1:**
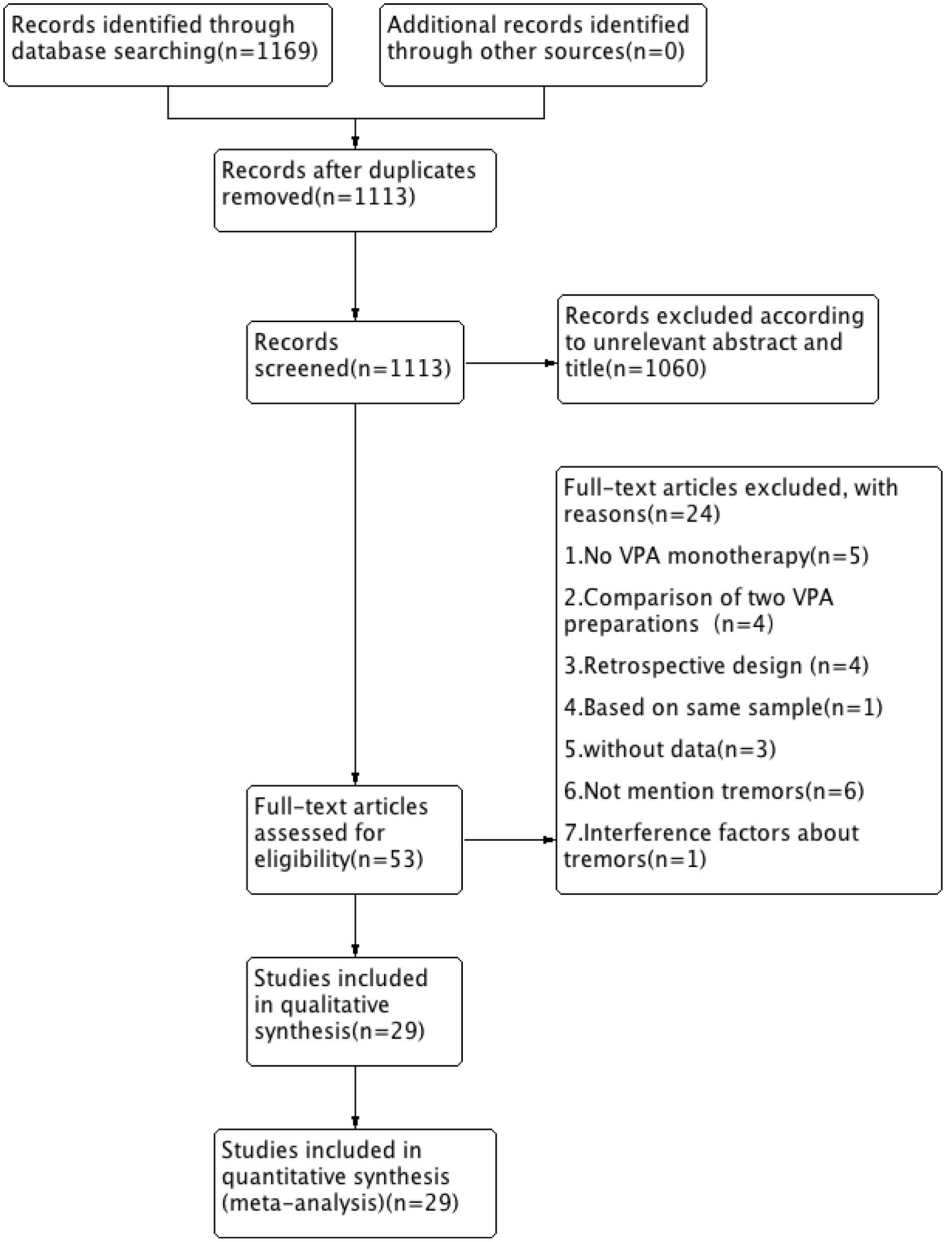
Flow chart of selection of studies about tremor in patients taking VPA therapy. VPA, valproic acid.

A total of 1,169 records were retrieved from the Embase, PubMed, and Cochrane library databases. As shown in Figure 1, after duplicates were removed, 1,113 articles remained. During our initial screening process, the titles and abstracts were read, and we eliminated reviews, meta-analyses, single case studies, case series, conference abstracts, commentaries, letters, and editorials. Fifty-three human clinical studies remained. After the full-text review, 24 studies were excluded. Finally, based on the inclusion criteria, 29 RCT studies (a total of 1,986 participants) that presented information on VPA-associated tremor were included in the evaluation of tremor incidence (Table 1).

In one study (23), three different doses of VPA were used and compared with a placebo, so we counted this study as three trials. In three studies (24–26), two different AEDs were compared to VPA, so we considered these three studies as six trials. Thus, the total number of comparisons was 34. Most trials were multicenter trials and recruited patients from the USA and Europe, seven trials were conducted in Iran, and other studies were conducted in various countries, such as Korea, Germany, Turkey, and China. These trials included different diseases, all of which were treated by VPA, including epilepsy (15), migraine headache (11), and bipolar disorder (3).

The sample size of the included trials ranged from 33 to 613, and the number of participants treated with VPA ranged from 16 to 244. The age range of the study patients was 6–88 years. The mean VPA dosage ranged from 400 to 2,099 mg/day. The trial performed by Mattson, was the only one in which a 2,099-mg/day dose was tested. The treatment period ranged from 0.75 to 45 months.

### Quality Assessment of Included Studies

The results of quality assessments of the included 29 studies are presented in Table 2.

**Table 2:** Risk of bias table of included studies.

**Study ID**	**Sequence-generation**	**Allocation-concealment**	**Blinding (participants, personnel)**	**Blind of assessors**	**Incomplete outcome data**	**Selective outcomes reporting**	**Other sources of bias**
Afshari et al. (19)	Low	Low	Low	Unclear	High	Low	Low
Biton et al. (27)	Low	Low	Low	Low	Low	Low	Low
Blumenfeld et al. (28)	Low	Low	Low	Unclear	Low	Low	Low
Bostani et al. (29)	Low	Low	Low	Unclear	Low	Low	Low
Calabrese et al. (30)	High	Low	Low	High	Low	Low	Low
Christe et al. (31)	High	Low	Low	Unclear	Low	Low	Low
Craig and Tallis (32)	Low	High	Low	Low	High	Low	Low
Fakhoury et al. (33)	High	High	High	High	Low	Low	Low
Hebrani et al. (34)	High	Low	Low	Low	High	Low	Unclear
Hesami et al. (20)	Low	Low	Low	Low	High	Low	Low
Klapper (23)	High	Low	Low	High	Low	Low	Unclear
Mathew et al. (35)	High	Low	Low	Unclear	Low	Low	Low
Mattson et al. (36)	Low	Low	Low	Low	Low	Low	Low
Nejad et al. (37)	High	High	High	Unclear	Low	Low	Unclear
Park et al. (38)	Low	High	High	High	Low	Low	Low
Privitera et al. (23)	Low	Low	Low	Unclear	Low	Low	Low
Richens et al. (39)	High	High	High	High	Low	Low	Unclear
Sarchielli et al. (40)	Low	Low	Low	Low	Low	Low	Low
Silberstein and Collins (41)	High	High	Low	Unclear	Low	Low	Unclear
So et al. (42)	High	Unclear	Low	Low	Low	Low	Low
Sobaniec et al. (43)	High	Unclear	Low	Unclear	Low	Low	High
Steinhoff et al. (25)	High	Low	High	High	Low	Low	High
Stephen et al. (44)	Low	High	High	Unclear	Low	Low	Low
Tabrizi et al. (45)	High	High	High	Unclear	Low	Low	High
Togha et al. (46)	Low	Low	Low	Low	Low	Low	High
Tohen et al. (47)	Low	Low	Low	Low	Low	Low	Low
Wheless et al. (26)	Low	Low	Low	High	Low	Low	High
Xu et al. (48)	Low	Low	Low	Low	High	Low	Low
Yurekli (49)	High	Low add	Low	High	Low	High	Low

*Low, low risk of bias; High, high risk of bias; Unclear, unclear risk of bias*.

### Incidence of Tremor

**Figure 2:**
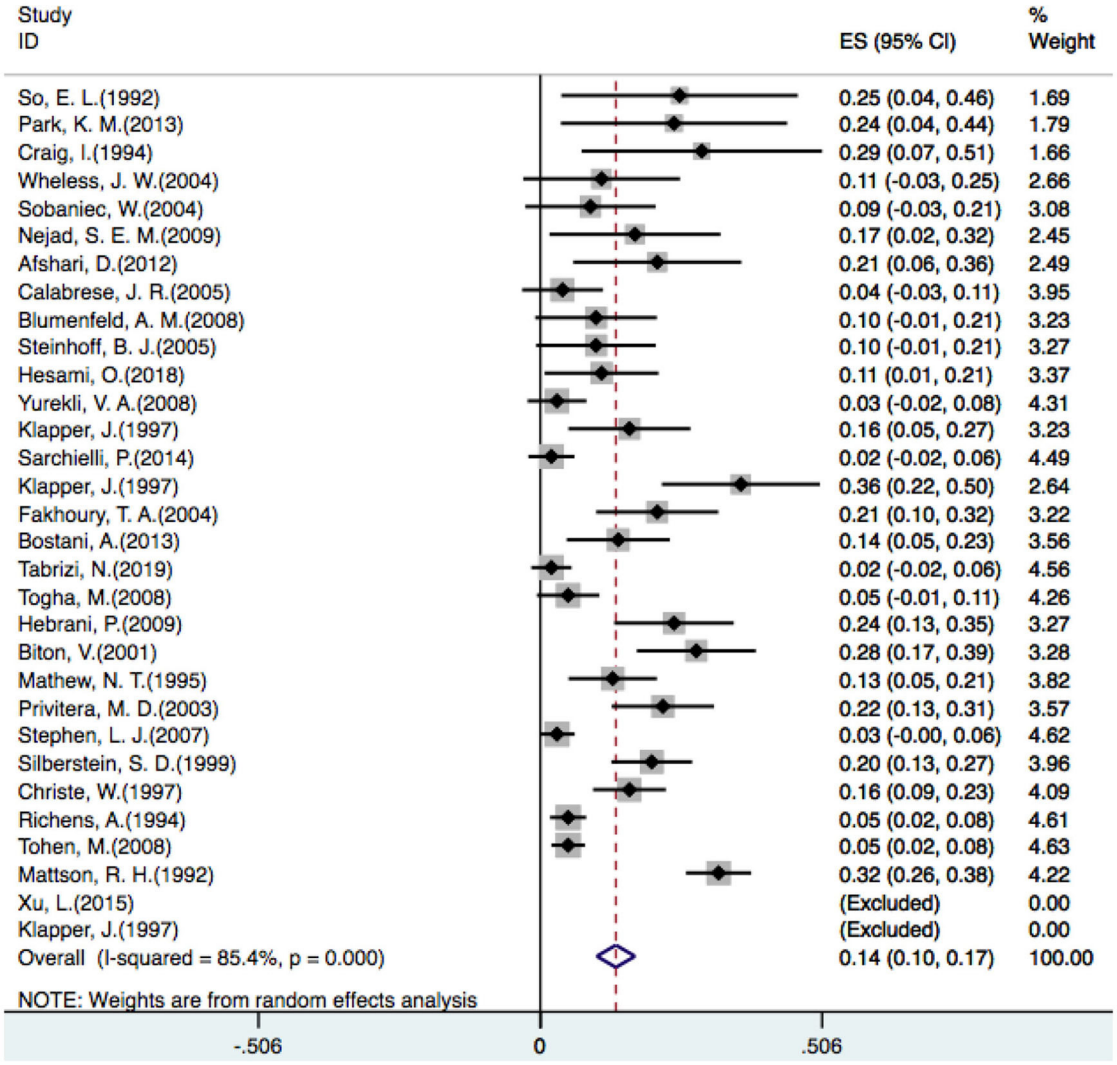
Pooled analysis of the overall incidence of VPA-related tremor. VPA, valproic acid.

In one placebo-controlled trial (23), three different doses of VPA were compared with a placebo, so we counted it as three trials. Additionally, two studies did not identify patients who developed tremors (23, 48), so 29 studies were included in our analysis. A random-effect model revealed that the overall incidence of tremor with VPA treatment was 14% [OR = 0.14, 95% CI (0.10–0.17)] (Figure 2).

### Comparison Between Various Drugs

**Figure 3:**
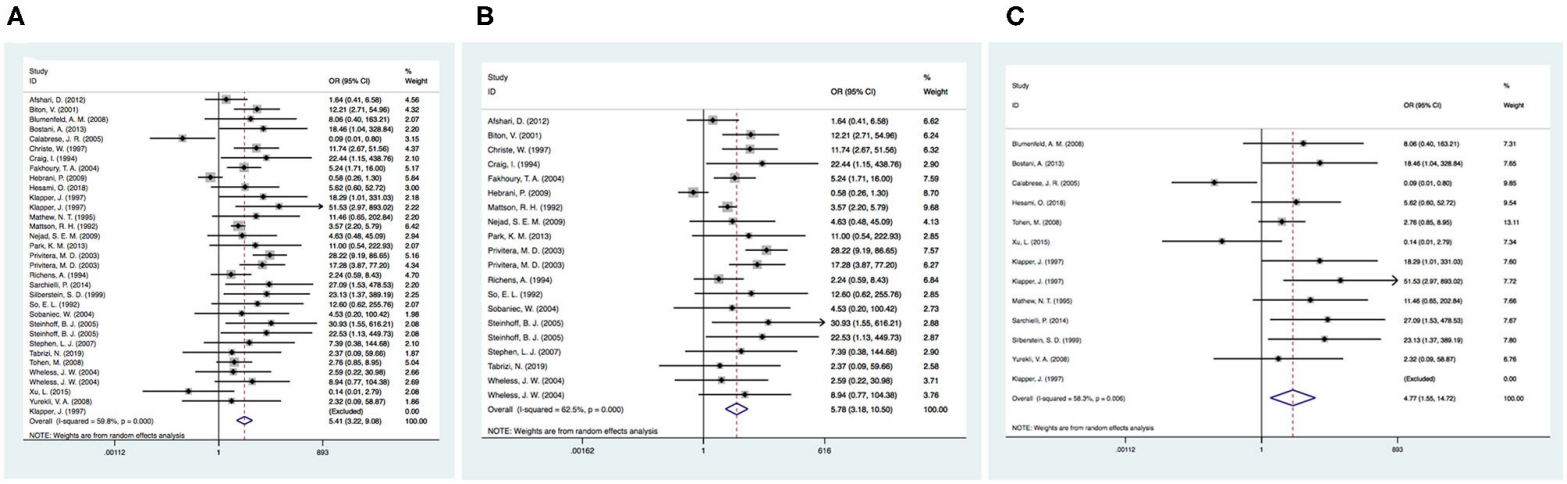
**(A)** The pooled OR of VPA-associated tremor compared with all other drugs. **(B)** The pooled OR of VPA-associated tremor compared with all other AEDs (except VPA), **(C)** The pooled OR of VPA-associated tremor compared with all other non-AEDs. AEDs, antiepileptic drugs; VPA, valproic acid.

**Figure 4:**
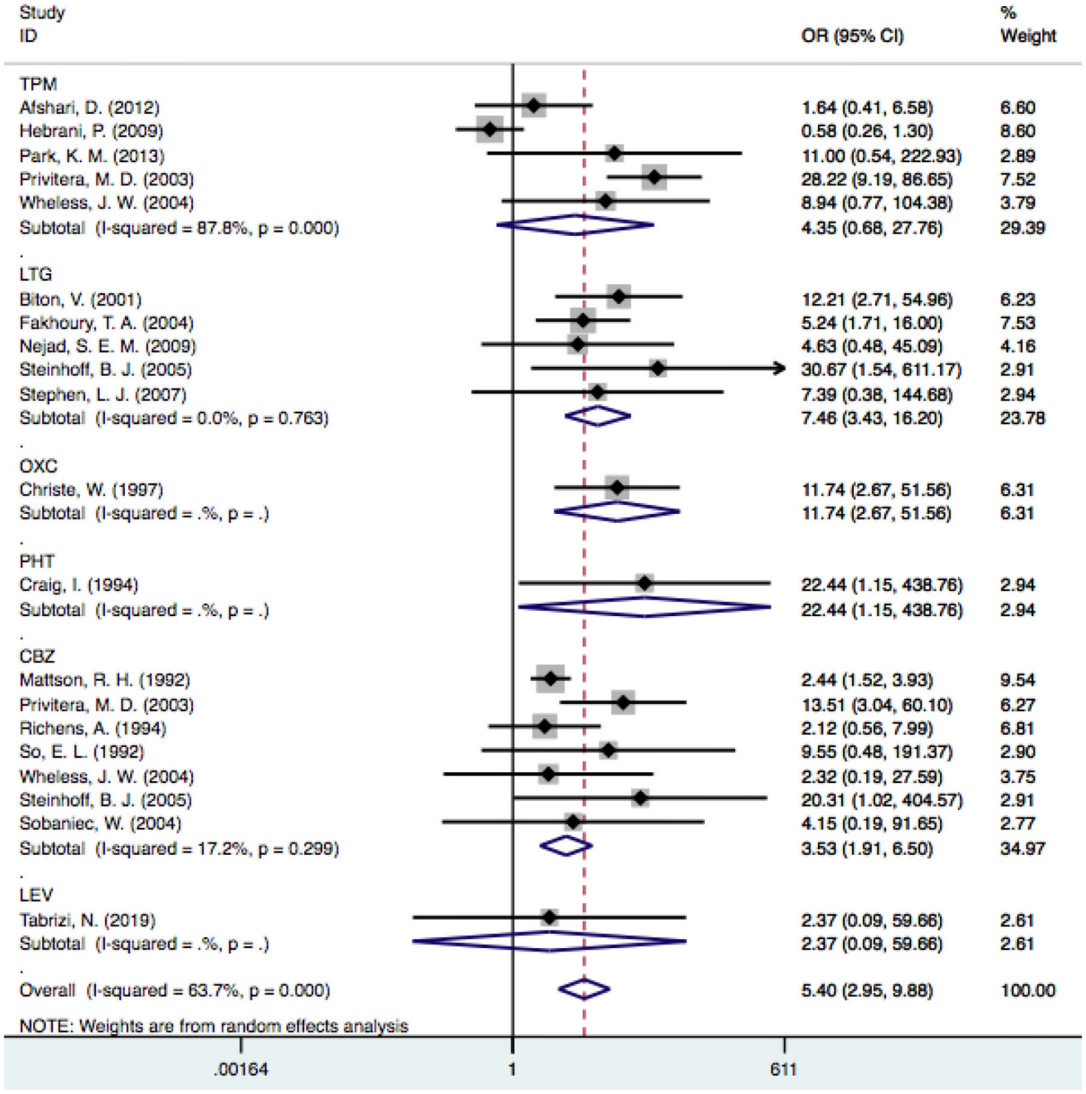
The pooled OR of VPA-associated tremor with other AEDs respectively. VPA, valproic acid; AEDs, antiepileptic drugs; TPM, topiramate; LTG, Lamotrigine; OXC, oxcarbazepine; PHT, phenytoin; CBZ, carbamazepine; LEV, levetiracetam.

Twenty-eight articles were included because one prospective study did not clearly determine the incidence of tremor in the control group (46). We independently evaluated the pooled ORs of VPA-induced tremor compared with tremor induced by all other drugs (other AEDs, other non-AEDs) to investigate the specific effect of VPA on tremors. According to our analysis, the use of VPA was significantly associated with an increased risk of tremor compared to that of the control group (Figure 3), including patients taking other drugs [28 articles, OR = 5.40, 95% CI (3.22–9.08)], other AEDs [17 articles, OR = 5.78, 95% CI (3.18–10.50)], and other non-AEDs [11 articles, OR = 4.77, 95% CI (1.55–14.72)]. In addition, we separately compared VPA to other AEDs and found a significant difference in the tremor risk between patients treated with VPA and other AEDs (Figure 4) [LTG OR = 7.46, 95% CI (3.43–16.20); CBZ OR = 3.53, 95% CI (1.91–6.50)]. However, there was no significant difference in the tremor risk between patients treated with VPA and TPM [OR = 4.35, 95% CI (0.681–27.76), *P* = 0.120]. There was only one article on OXC, LEV, and PHT. These comparisons exhibited heterogeneity, so random-effect models were used.

### Subgroup Analysis

The former comparisons of VPA-induced tremor compared with all drugs exhibited heterogeneity, and we considered that the sample size, VPA dosage, and follow-up time influenced the outcomes. Therefore, subgroup analysis was performed according to the following three factors.

### Sample Size

**Figure 5:**
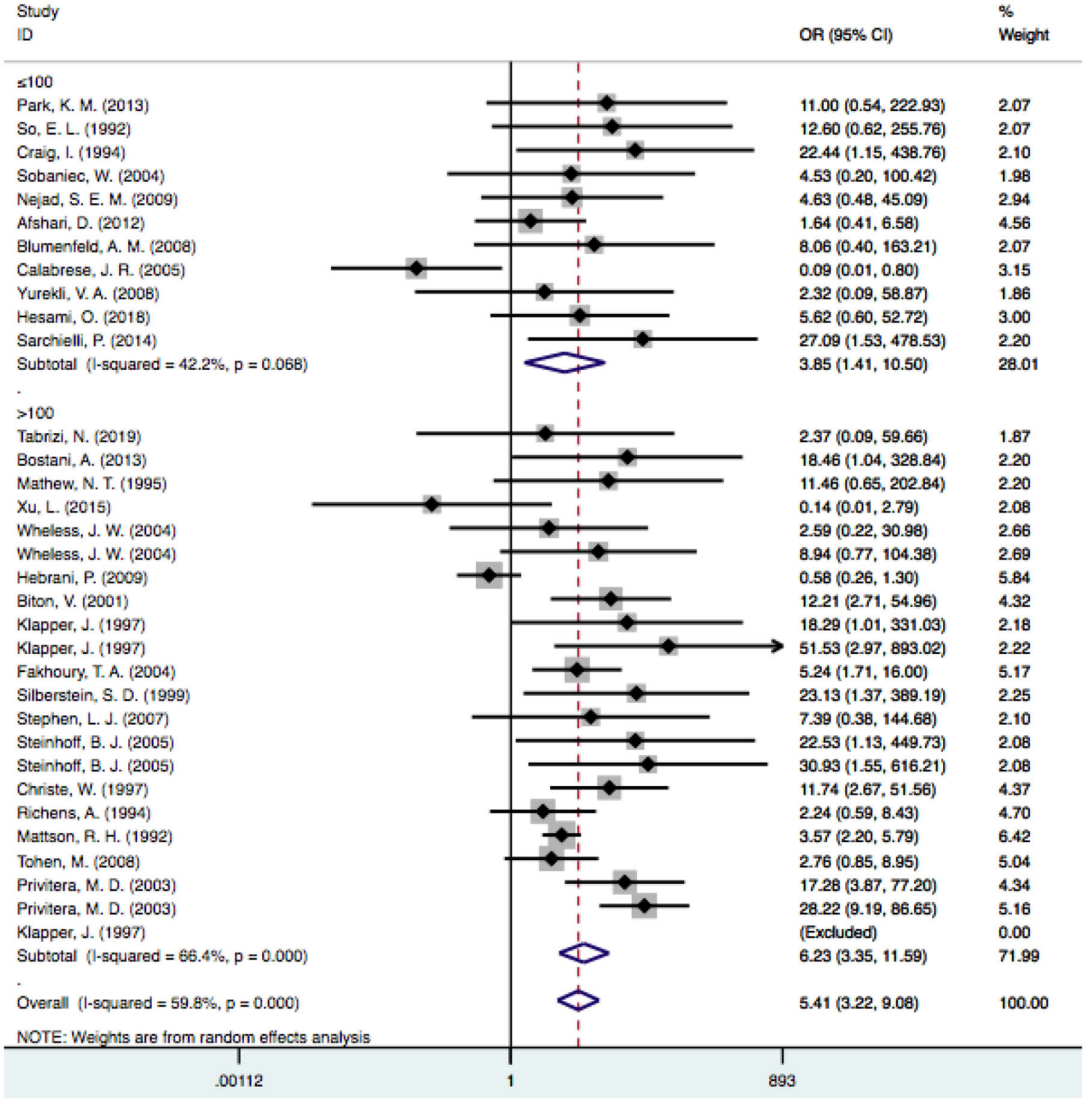
Subgroup analysis of VPA-associated tremor compared with other drugs according to the sample size. VPA, valproic acid.

In the subgroup analysis performed by sample size, we used a threshold of 100 patients. It was noted that the risk of VPA-associated tremors in the studies with more than 100 patients differed significantly from the risk of tremors associated with other drugs [OR = 6.23, 95% CI (3.35–11.59)], and this significant difference persisted in the groups with fewer than 100 patients [OR = 3.85, 95% CI (1.41–10.50)] (Figure 5). Among the studies with larger sample sizes, the risk of VPA-related tremors was larger than that in the studies with a small sample size, which reflected the stability of our meta-analysis. Neither group exhibited a significant difference in heterogeneity (I^2^ = 42.2% and I^2^ = 66.4%, respectively).

### VPA Doses Used

**Figure 6:**
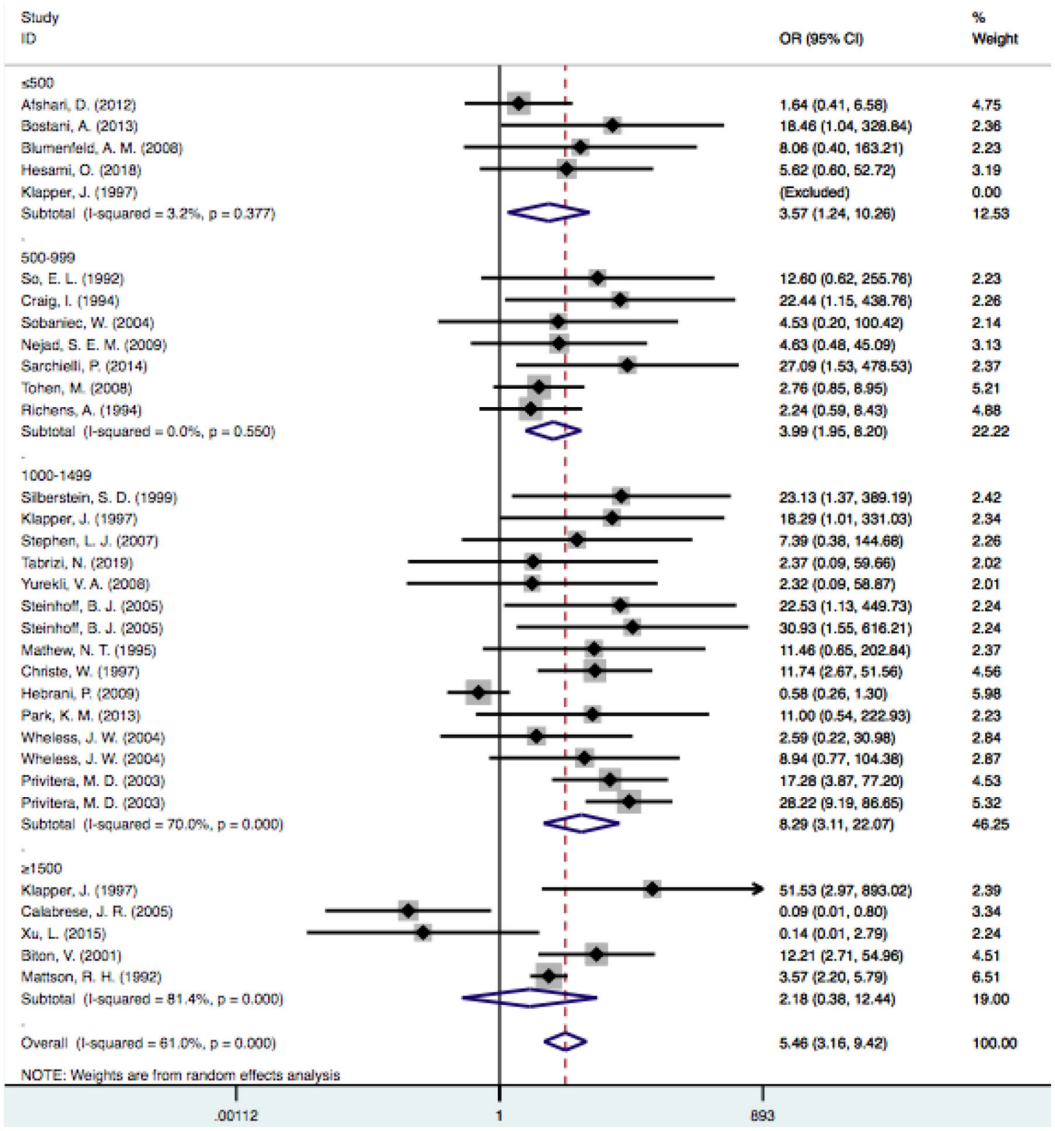
Subgroup analysis of VPA-associated tremor compared with other drugs according to the different drug dose. VPA, valproic acid.

In the subgroup analysis of the doses, we excluded one study because it did not clearly provide the mean VPA dosage (33). According to the dosage used, we divided the patients into four subgroups: the ≤ 500-mg/d, 500–999-mg/d, 1,000–1,499-mg/d, and ≥1,500-mg/d subgroups. When the dose reached ≥1,500 mg/d, the level of heterogeneity was considered significant (I^2^ = 81.4% vs. I^2^ = 59.8%). Considering that dose-related factors may influence the results, we excluded articles that reported doses >1,500 mg/d (23, 27, 30, 36, 48), but the level of heterogeneity did not decrease significantly (I^2^: 54.6 vs. 59.8%). Therefore, we did not consider studies with a mean dose >1,500 mg/d as a source of heterogeneity. The pooled estimate for VPA-related tremors was significantly different from that of tremors related to other drugs at doses of 500 mg/d [OR = 3.57, 95% CI (1.24–10.26)], 500–999 mg/d [OR = 3.99, 95% CI (1.95–8.20)] and 1,000–1,499 mg/d [OR = 8.82, 95% CI (3.25–23.94)], and there were no statistically significant differences regarding other drugs at doses ≥1,500 mg/d [OR = 2.17, 95% CI (0.38–12.44); *P* = 0.381] (Figure 6).

### Duration Times

**Figure 7:**
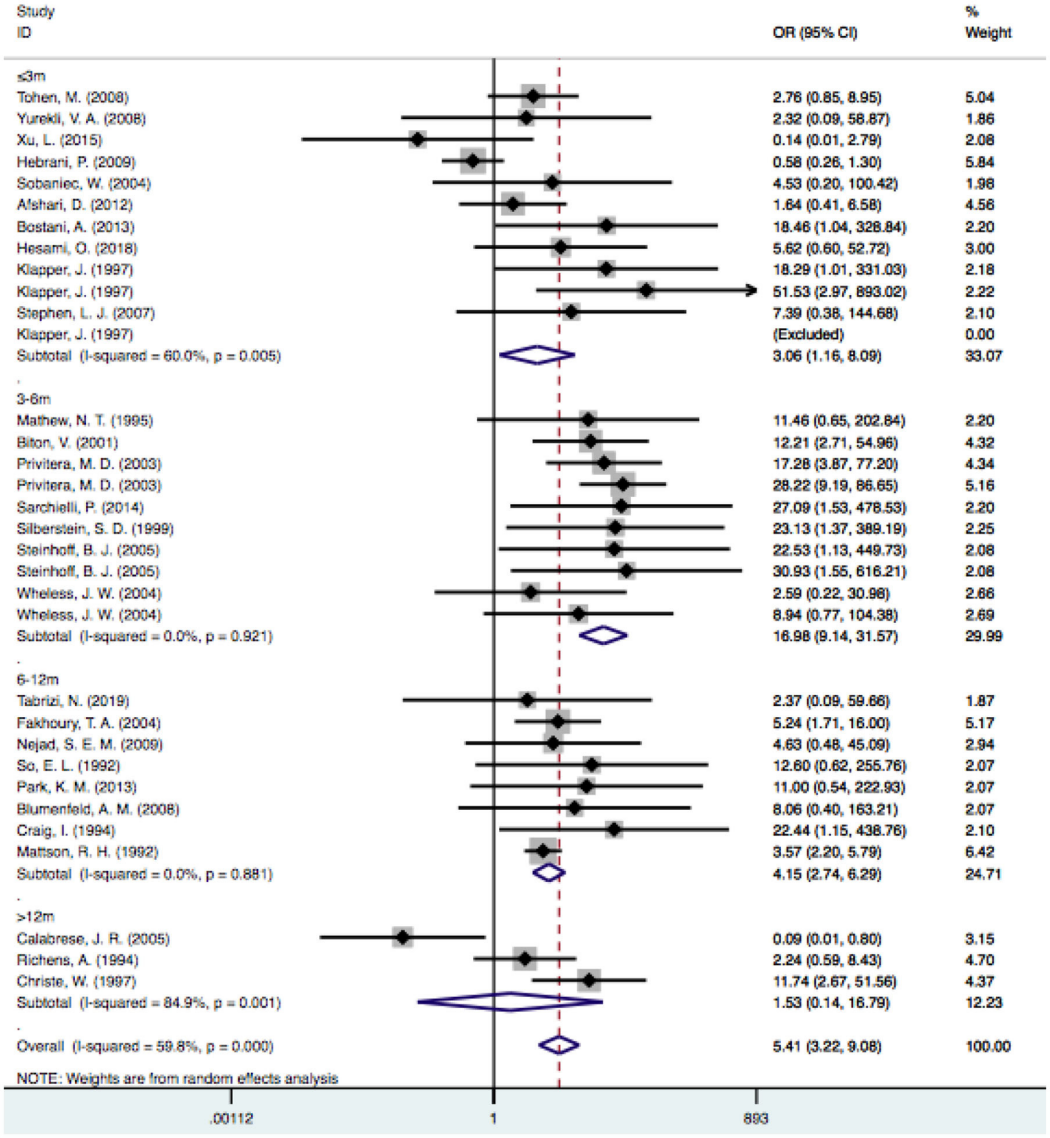
Subgroup analysis of VPA-associated tremor compared with other drugs according to the duration time. VPA, valproic acid.

Based on the follow-up time, the pooled estimate of VPA-associated tremor was statistically significantly higher than that of tremors associated with other drugs at duration times of ≤ 3 months [OR = 3.06, 95% CI (1.16–8.09)], 3–6 months [OR = 16.98, 95% CI (9.14–31.57)] and 6–12 months [OR = 4.15, 95% CI (2.74–6.29)]. VPA-induced tremor had a higher incidence during treatment durations of 6–12 months than during durations of ≤ 3 and 3–6 months. However, the risk of VPA-related tremor was not significantly different for durations >12 months [OR = 1.53, 95% CI (0.14–16.79); *P*=0.730] (Figure 7).

### Sensitivity Analysis

**Figure 8:**
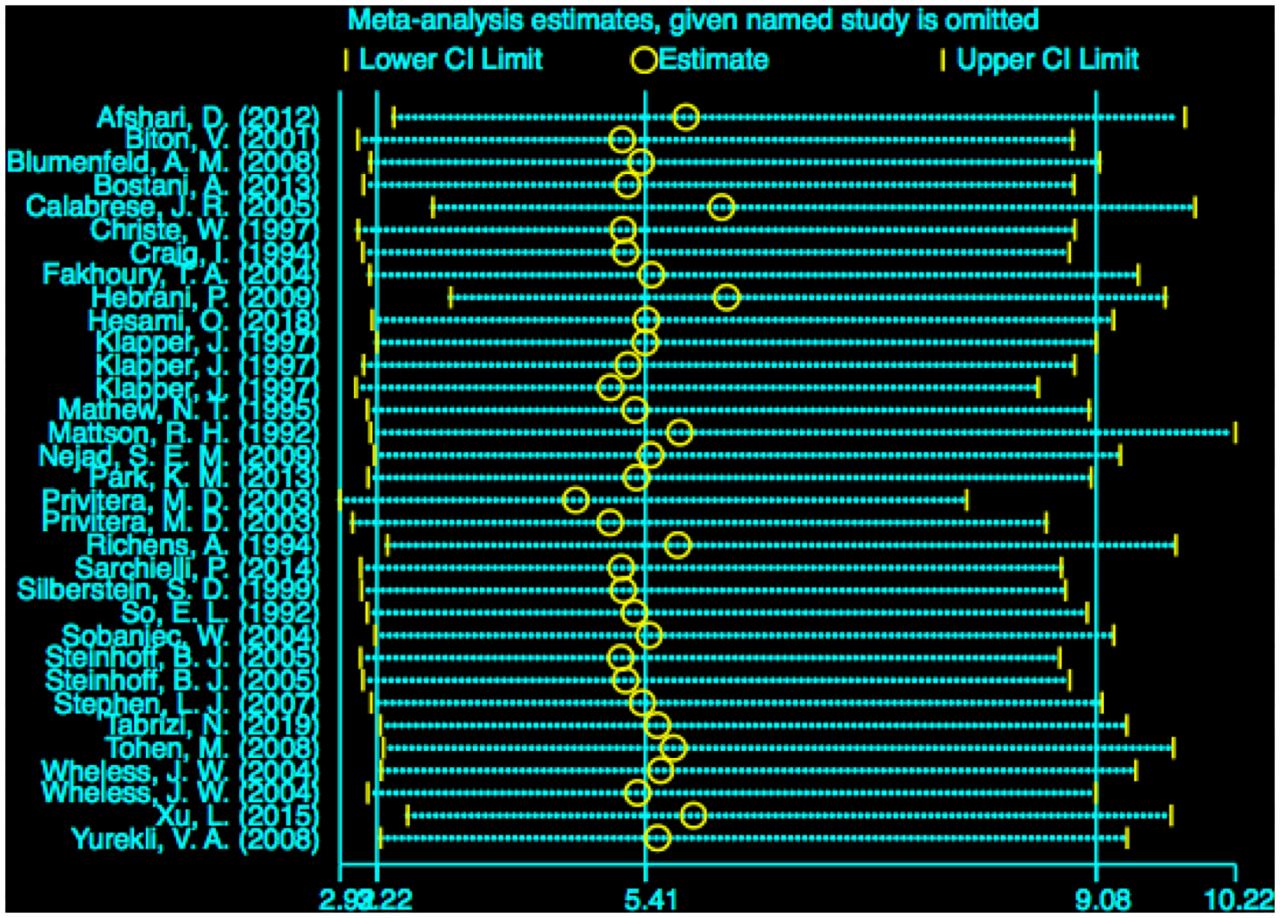
Sensitivity analysis of VPA-associated tremor compared with other drugs. VPA, valproic acid.

The stability of the pooled estimate was assessed by excluding ineligible studies one by one and reconducting the analysis. The outcomes of VPA-associated tremors were not significantly affected, indicating that the results of our analysis were stable (Figure 8).

### Publication Bias

**Figure 9:**
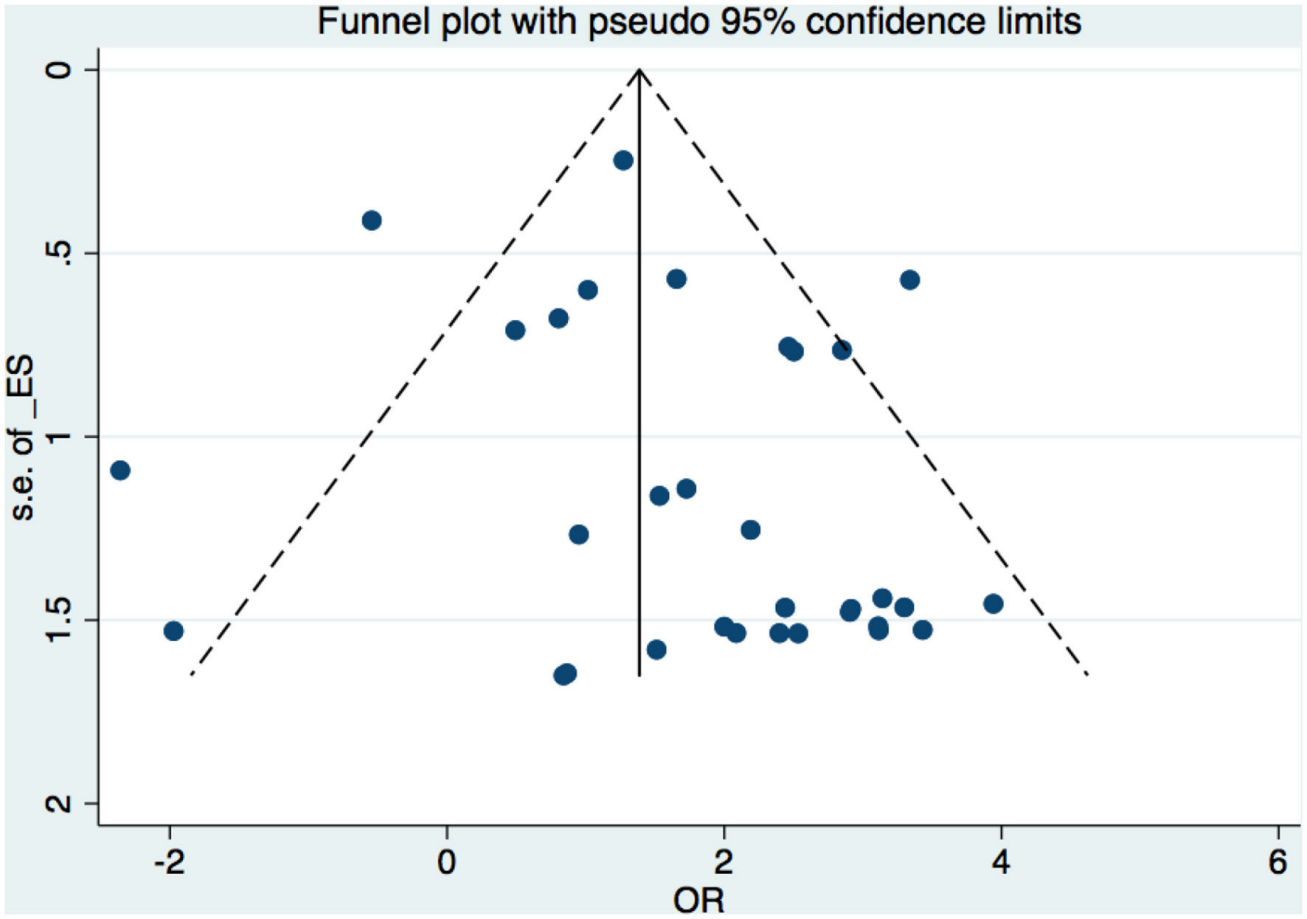
Funnel plot of VPA-associated tremor compared with other drugs. VPA, valproic acid.

**Figure 10:**
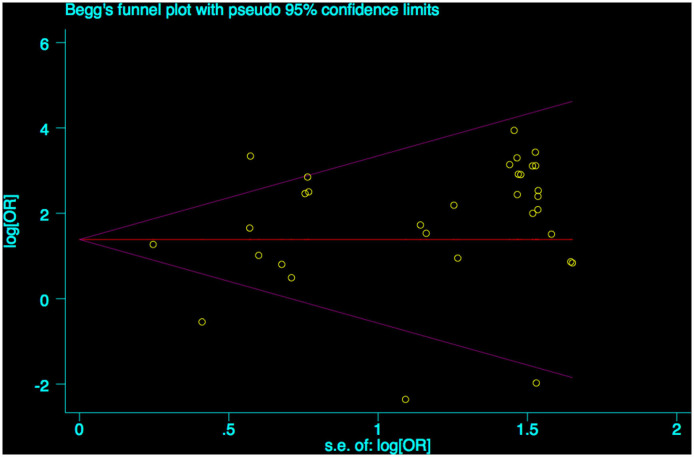
Begg's funnel plot test of publication bias for VPA-associated tremor. VPA, valproic acid.

**Figure 11:**
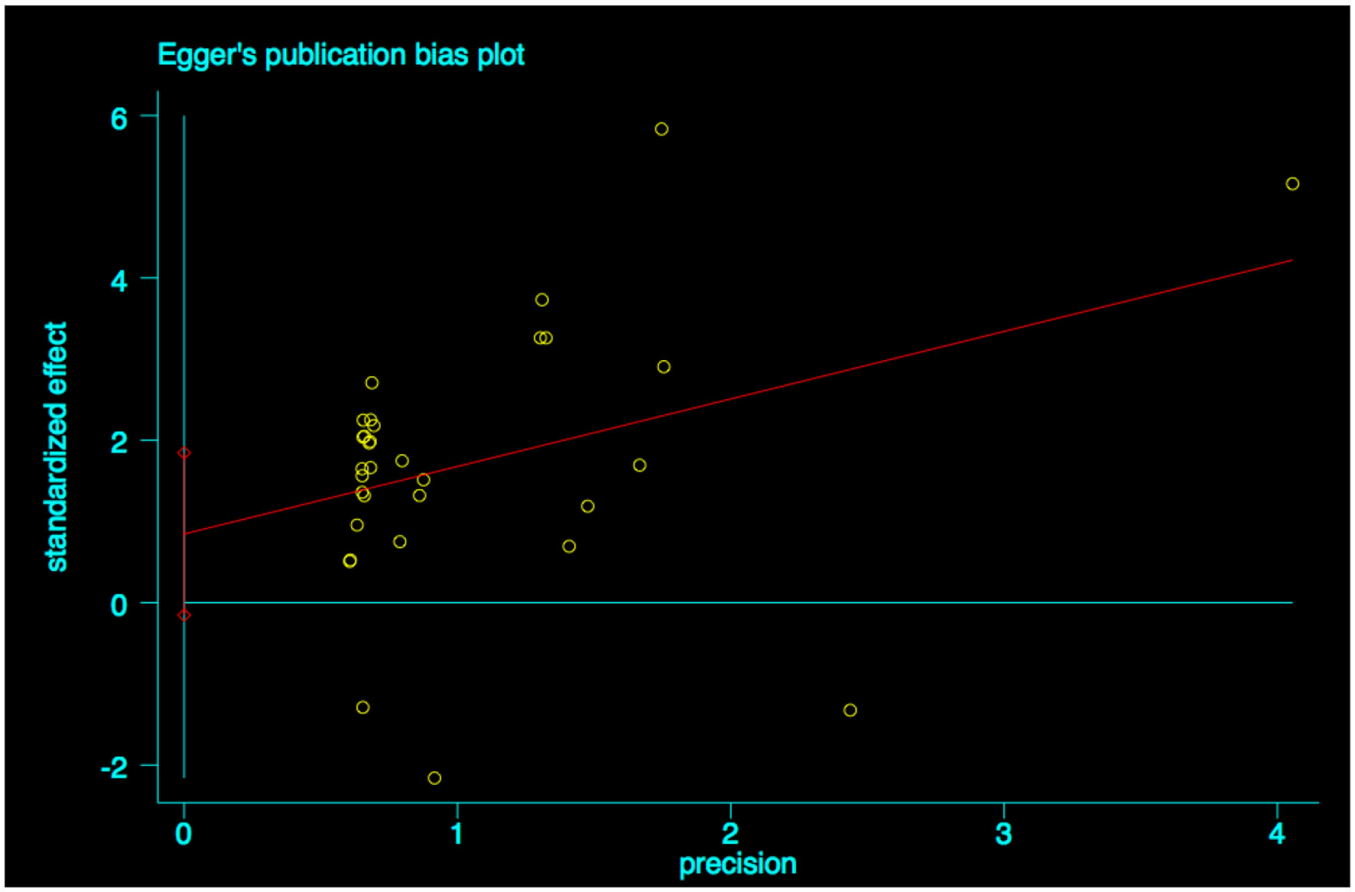
Egger's linear regression test of publication bias for VPA-associated tremor. VPA, valproic acid.

A funnel plot was used to detect bias in these studies, and the plot seemed to be asymmetrically distributed (Figure 9). Begg's test and Egger's test were conducted, and both tests indicated a lack of publication bias for VPA-associated tremors compared to tremors associated with other drugs (*P* = 0.922, *P* = 0.094, Figures 10, 11).

## Discussion

According to previous studies, the evidence for VPA-induced tremor has been well documented. In epilepsy patients, VPA-related tremor is presumed to be one of the most common side effects, with an incidence of 6–64% (36, 39, 50). The mechanism underlying VPA-induced tremors is not completely understood; it has been suggested that the occurrence of tremors with VPA could be explained by the marked changes in the GABA synthesis rate in the substantia nigra and corpus striatum (51), and disturbances of the GABAergic pathways in the basal ganglia system may result in DA inhibition and subsequent changes in catecholamine (NE and E) concentrations (52–54). There is considerable evidence indicating that VPA increases the concentration of the inhibitory neurotransmitter gamma-aminobutyric acid (GABA), and this mechanism is thought to be a major cause of tremor (55, 56). The increase in transmitters occurs mainly through the following two pathways: (1) inhibition of the activation of glutamate transaminase and succinic acid dehydrogenase to reduce its metabolism and (2) activation and increase glutamate decarboxylase synthesis. At the same time, VPA increased GABA receptor-mediated hyperylation and inhibited the activation of the N-methyl-aspartic acid receptor. The factors of tremor severity may include the patient's sex and age and the dosage and formulations of VPA (55, 56). VPA often exaggerates familial tremor, and this sort of tremor becomes more prevalent and more noticeable as people age. However, whether the relation of VPA-associated tremor with aging is partly a consequence of previously unrecognized essential tremor cases worsening is unknown.

This meta-analysis provides a valuable and relatively complete description of the incidence and risk of VPA-associated tremor compared to tremor associated with other drugs. Our study included 29 RCT trials, and the overall incidence of tremor in patients treated with VPA was estimated to be 14%. According to Farkas et al. (57), the true incidence of VPA-induced tremor may be underestimated, as quantitative methods suggest that motor disturbances may predate symptoms of tremor. Tremor is probably not the first appearing motor adverse effect of valproate treatment. Quantitative methods might reveal a higher incidence of valproate-related motor disturbances than is currently considered (57). Furthermore, we observed a significant difference among patients treated with VPA and all other drugs (other AEDs, non-AEDs). Patients taking VPA had an approximately 4.5-fold higher (17.5 vs. 3.9%) risk of developing tremor than did patients taking other AEDs and an approximately 5-fold higher (11.7 vs. 2.5%) risk than did patients taking other non-AEDs. Compared to a single AED, the risk of VPA-induced tremor was greater than that of LTG- (7.47 times) and CBZ-induced tremor (3.53 times). Since only a few included studies reported the incidence of tremor associated with other AEDs, we were unable to calculate the pooled estimates. This meta-analysis could provide insights into alternative antiepileptic drugs to VPA.

Subgroup analyses were conducted to evaluate the impact of different drug dosages. VPA-induced tremor was found to be dose-related, but the means of the serum levels of VPA were within the normal therapeutic ranges (54). Patients with high blood VPA concentrations tend to develop adverse effects (58). VPA-induced tremor was reported to be reversible upon reduction or withdrawal of the drug and worsened as the dose increased (18, 57). Based on our meta-analysis, we divided the included trials into four groups (≤500 mg/d, 500–999 mg/d, 1,000–1,499 mg/d, ≥1,500 mg/d) according to the mean daily drug dose, and we drew a similar conclusion regarding VPA dose-related. A dose-dependent effect of VPA-related tremor was observed when the four dose groups were compared. Patients taking doses of 1,000–1,500 mg/d had an increased risk of developing tremor compared to patients who were administered both doses ≤ 500 mg/d and doses ranging from 500 to 999 mg/d. It is worth mentioning that VPA is often supplied as a sodium salt, and the molecular weight differed across the included studies. Therefore, future research should provide a more detailed description of the VPA dose.

Medication retention includes all possible reasons for effectiveness and intolerability (59), especially due to the side-effect profile (60). If patients experience VPA-related tremors, clinicians should consider whether the VPA dose is within the therapeutic dosage and whether it is possible to reduce the VPA dose. It may be a wise approach to choose another drug instead of VPA if the effect cannot be maintained after reducing the dosage.

According to the reports of Karas et al. (16), tremors occur as early as within 1 month of therapy. Tremors are present and exacerbated by intentional positions, and as the treatment continues, tremors gradually appear or become more severe. Our meta-analysis confirmed this result. We also divided the studies into four groups according to the follow-up time (≤ 3 m, 3–6 m, 6–12 m, >12 m); when we pooled the results of studies that followed up patients for 6–12 months, the risk of tremor was higher than that in studies that followed up patients for <3 months or from 3 to 6 months. This finding reminds us that the risk of VPA-related tremor may be time-related. At present, no studies have clearly examined the relation between the duration of treatment and risk of tremor. Hence, these data may lay the foundation for future studies, which may be profoundly meaningful for clinical therapy.

Due to the comprehensive search strategy used, high-quality RCTs were included, and the statistical heterogeneity of the outcomes in the pooled estimate analyses and stable sensitivity analysis results suggested our results have good reliability. However, our study also had several limitations. First, in the 29 trials, only 1,986 participants were treated with VPA, and among them, only a small number experienced tremor due to VPA therapy. Second, all the included studies were RCTs, but not all of the studies employed a double-blind design, so the quality of some studies was not relatively high. Third, VPA-related tremor is more common in elderly people and women, but relevant data were unavailable for us to perform a pooled estimation and confirm this conclusion. In addition, the number of the included studies and the AEDs included in comparisons were relatively small; therefore, we need to be cautious when drawing general conclusions.

## Conclusion

VPA-associated tremor is more common than many clinicians have realized, with an overall incidence of 14%. VPA poses a higher risk of tremor than do other AEDs. VPA-induced tremor does depend on a dose <1,500 mg/d, and reducing the drug dose can reduce the risk. If the effect cannot be maintained, we recommend replacing VPA with other drugs (such as LTG or CBZ). Additionally, VPA-induced tremor can occur in every period after VPA treatment and gradually worsen over time. Patients taking VPA have a higher risk of experiencing tremor than do patients taking other drugs within 12 months, and patients should be advised about the risk. In summary, these results support more large population-based studies, and longer duration clinical trials are urgently needed to confirm whether VPA increases the risk of developing tremor compared to other drugs and whether the risk is related to dose and duration treatment times.

## Data Availability Statement

The original contributions generated for the study are included in the article/supplementary material, further inquiries can be directed to the corresponding author/s.

## Author Contributions

CqZ and HbS performed the experiments. BmH and HbS supervised the data collection. MlH and HbS wrote the initial draft. All authors contributed to the article and approved the submitted version.

## Conflict of Interest

The authors declare that the research was conducted in the absence of any commercial or financial relationships that could be construed as a potential conflict of interest.

## Abbreviations

VPA: valproic aid
RCTs: randomized controlled trials
AEDs: antiepileptic drugs
FDA: Food and Drug Administration
PTSD: posttraumatic stress disorder
CBZ: carbamazepine
TPM: topiramate
VGT: vigabatrin
LTG: lamotrigine
TGB: gabapentin
ORs: odds ratios
CIs: confidence intervals.
